# Phenolic compounds apigenin, hesperidin and kaempferol reduce in vitro lipid accumulation in human adipocytes

**DOI:** 10.1186/s12967-017-1343-0

**Published:** 2017-11-21

**Authors:** Saioa Gómez-Zorita, Arrate Lasa, Naiara Abendaño, Alfredo Fernández-Quintela, Andrea Mosqueda-Solís, Maria Pilar Garcia-Sobreviela, Jose M. Arbonés-Mainar, Maria P. Portillo

**Affiliations:** 1 Nutrition and Obesity Group, Department of Nutrition and Food Science and Lucio Lascaray Research Center, University of the Basque Country (UPV/EHU), Vitoria, Spain; 2 Adipocyte and Fat Biology Laboratory (AdipoFat), Unidad de Investigación Traslacional, Instituto Aragonés de Ciencias de la Salud (IACS), Instituto de Investigación Sanitaria (IIS) Aragón, Zaragoza, Spain; 3 CIBER Fisiopatología Obesidad y Nutrición (CIBERobn), Instituto de Salud Carlos III, Madrid, Spain

**Keywords:** Adipocytes, Apigenin, Hesperidin, Kaempferol, Obesity, Human mesenchymal stem cells

## Abstract

**Background:**

Adipocytes derived from human mesenchymal stem cells (MSCs) are widely used to investigate adipogenesis. Taking into account both the novelty of these MSCs and the scarcity of studies focused on the effects of phenolic compounds, the aim of the present study was to analyze the effect of apigenin, hesperidin and kaempferol on pre-adipocyte and mature adipocytes derived from this type of cells. In addition, the expression of genes involved in TG accumulation was also measured.

**Methods:**

Pre-adipocytes were cultured from day 0 to day 8 and mature adipocytes for 48 h with the polyphenols at doses of 1, 10 and 25 µM.

**Results:**

Apigenin did not show an anti-adipogenic action. Pre-adipocytes treated with hesperidin and kaempferol showed reduced TG content at the three experimental doses. Apigenin did not modify the expression of the main adipogenic genes (*c/ebpβ, c/ebpα, pparγ* and *srebp1c*), hesperidin inhibited genes involved in the three phases of adipogenesis (*c/ebpβ, srebp1c* and *perilipin*) and kaempferol reduced *c/ebpβ*. In mature adipocytes, the three polyphenols reduced TG accumulation at the dose of 25 µM, but not at lower doses. All compounds increased mRNA levels of *atgl*. Apigenin and hesperidin decreased *fasn* expression. The present study shows the anti-adipogenic effect and delipidating effects of apigenin, hesperidin and kaempferol in human adipocytes derived from hMSCs. While hesperidin blocks all the stages of adipogenesis, kaempferol only inhibits the early stage. Regarding mature adipocytes, the three compounds reduce TG accumulation by activating, at least in part, lipolysis, and in the case of hesperidin and apigenin, also by reducing lipogenesis.

**Conclusions:**

The present study shows for the first time the anti-adipogenic effect and delipidating effect of apigenin, hesperidin and kaempferol in human adipocytes derived from MSCs for the first time.

## Background

Overweight and obesity are considered a serious threat to public health, due to their high prevalence in our society and their association with co-morbidities such as type 2 diabetes, hypertension and cardiovascular diseases [1, 2].

In vitro studies performed using adipocytes derived from human mesenchymal stem cells (MSCs) represent a good method to analyze the two main processes that lead to adipose tissue increase and thus, to obesity development: hypertrophy (increased adipocyte size) and hyperplasia (increased adipocyte number) [1, 2]. MSCs are fibroblastoid multipotent adult stem cells with a high capacity for self-renewal. These cells have been isolated from several human tissues, such as bone marrow, adipose tissue, umbilical cord matrix, tendon, lung and periosteum, among others [3]. MSCs become adipoblasts, which subsequently commit to pre-adipocytes. Thereafter, upon adipogenic stimuli, pre-adipocytes undergo terminal differentiation into mature adipocytes [4]. Recently, it has been suggested that MSCs are a main source of adipocyte generation. Therefore, the biology of MSCs is studied in this work, as is the possible roles of MSCs in managing different components of metabolic syndrome [5–7].

In recent years, a great deal of attention has been paid to new active biomolecules that could be effective in preventing or treating obesity and its co-morbidities. Numerous studies have been carried out with phenolic compounds, which are members of a very large family of plant-derived compounds in the form of a wide variety of chemical structures [8]. Of these, resveratrol, quercetin and epigallocatechin have been shown to prevent fat accumulation in adipocytes through different mechanisms: by adipogenesis and lipogenesis inhibition as well as by lipolysis and fatty acid oxidation stimulation [9–11]. It must be pointed out that the majority of these studies have been performed in 3T3-L1 or mouse and rat primary adipocytes. However, studies demonstrating the effect of these phenolic compounds on human adipocytes are scarce to date [12–19]. Furthermore, important differences in adipocyte function exist among species, which complicates the extrapolation of results from murine adipocytes to human adipocytes [20]. Consequently, human MSCs provide an important alternative model system which represents a valuable instrument for experimental human fat cell investigation [21, 22].

Taking into account the novelty of these MSCs as an in vitro model for the study of obesity, and the few studies performed on human cells with phenolic compounds, the aim of the present study was to analyze the effect of three polyphenols (two flavonoids: apigenin and hesperidin; and one non-flavonoid: kaempferol). These polyphenols were previously studied in 3T3-L1 cells in our laboratory [23], in pre-adipocyte and mature adipocytes derived from this type of cells. In addition, the expression of genes involved in TG accumulation was also measured.

## Methods

### Cell samples

Human mesenchymal fat cells (hMSCs) were obtained from subcutaneous abdominal fat from a 59 year-old man with overweight and without type 2 diabetes mellitus, hypertension or dyslipidemia or obesity, as previously described [24], and approved by Clinical Investigation Ethics Committee of Aragon (Acta 11/2013).

### Experimental design

Cells were cultured until confluence in DMEM 1 g/L glucose (Lonza, Verviers, Belgium) containing 10% fetal bovine serum (FBS), 1 mM sodium pyruvate, 4 mM glutamine, 1% penicillin/streptomycin. Differentiation was induced 2 days post-confluence (designated as day 0) with DMEM 4.5 g/L glucose (Lonza, Verviers, Belgium) supplemented with 10% FBS, 1 μM dexamethasone, 0.5 mM isobutylmethylxanthine, 1 μM rosiglitazone and 1.67 μM human insulin. This medium was changed every 72 h. At day 6, cells were incubated in a DMEM 4.5 g/L glucose medium (Lonza, Verviers, Belgium) containing 1 mM sodium pyruvate, 4 mM glutamine, 10% FBS, 1% penicillin/streptomycin and this medium was changed every 72 h until cells were treated. Cells were maintained at 37 °C in a humidified 5% CO_2_ atmosphere. Each experiment was performed three times.

Differentiating cells were grown in 6-well plates and incubated together with either 0.1% ethanol (95%) (control group) or with apigenin (Extrasynthese, Lyon, France), hesperidin (Extrasynthese, Lyon, France) or with kaempferol (Genay, Lyon, France) at 1, 10 or 25 µM (diluted in 95% ethanol). In a first experiment, on day 3 cells treated with phenolic compounds were used at 25 µM for TG content determination and RNA extraction. In a second experiment, on day 8 cells treated with phenolic compounds were used at 1, 10 or 25 µM for TG content determination and cells treated with 25 µM were used for RNA extraction and cytotoxicity determination.

Mature adipocytes grown in 6-well plates were also incubated with either 0.1% ethanol (95%) (control group) or with apigenin, hesperidin or kaempferol at 1, 10 or 25 μM (diluted in 95% ethanol) on day 12 after differentiation. After 48 h of treatment, supernatant was removed and cells were used for triacylglycerol (TG) determination and RNA extraction.

### Triacylglycerol content

For TG determination, cells were washed with phosphate buffer saline (PBS) and incubated 3 times with 800 µL of hexane/isopropanol (2:1). The total volume was evaporated under nitrogen, and the pellet was resuspended in 100 μL of Triton X-100 in 1% distilled water. Afterward, TG were solubilized by a sonicator, and the content was measured using infinity triglycerides reagent (Spinreact, Girona, Spain). For protein determination, cells were lysed in 0.3 N NaOH and 0.1% SDS. Protein measurements were performed using the BCA reagent (Thermo Fischer Scientific, Rockford, USA).

### Extraction and analysis of RNA and quantification by real-time PCR

RNA samples were extracted from cells treated with 25 µM of each phenolic compound by using Trizol (Invitrogen, Carlsbad, CA, USA), according to the manufacturer’s instructions. The integrity of the RNA extracted from all samples was verified and quantified using a RNA 6000 Nano Assay (Thermo Scientific, Wilmington, DE, USA). RNA samples were then treated with DNase I kit (Applied Biosystems Inc., Foster City, CA, USA) to remove any contamination with genomic DNA.

One microgram of total RNA in a total reaction volume of 20 μL was reverse transcribed using the iScript cDNA Kit (Applied Biosystems Inc., Foster City, CA, USA) according to the manufacturer’s protocols. Reactions were incubated initially at 25 °C for 10 min and subsequently at 37 °C for 120 min and at 85 °C for 5 min.

Relative mRNA levels were quantified using real-time PCR with an iCycler MyiQ Real Time PCR Detection System (Bio-Rad, Hercules, CA, USA). 18S mRNA levels were similarly measured and served as the reference gene. The PCR reagent mixture consisted of 4.75 μL of each diluted cDNA. SYBR Green Master Mix (Applied Biosystems, Foster City, CA, USA) and of the upstream and downstream primers (300 nM). Specific primers for c/*ebpβ* (CCAAT/enhancer-binding protein beta), *c/ebpα* (CCAAT/enhancer-binding protein alpha), *pparγ* (peroxisome proliferator factor gamma), *srebp1c* (sterol regulatory element-binding protein 1c), *acc* (acetyl-CoA carboxylase), *perilipin*, *scd1* (stearoyl-CoA desaturase 1), *atgl* (adipose triglyceride lipase), *hsl* (hormone sensitive lipase), *fasn* (fatty acid synthase) and *dgat* (diglyceride acyltransferase) were synthesized commercially and the sequences are shown in Table 1.

**Table 1:** Primer sequences for PCR amplification of each gene studied

	Sense primer	Antisense primer
*c/ebpβ*	5′-GGCAGCACCACGACTTCCT-3′	5′-CGCCCCAGGCTCACGTAG-3′
*c/ebpα*	5′-AGGGTCTCTAGTTCCACGCC-3′	5′-CAAGGGGAAGCCCAGCCTATA-3′
*pparγ*	5′-TAGATGACAGCGACTTGGCAATAT-3′	5′-GAATGTCTTCAATGGGCTTCACA-3′
*srebp1c*	5′-ACGCCCCACTTCATCAAGG-3′	5′-ACTGTTGCCAAGATGGTTCCG-3′
*acc*	5′-CATCAGCAGAGACTACGTCCTCAA-3′	5′-CATGGCAACCTCTGGATTGG-3′
*perilipin*	5′-TGGAGACTGAGGAGAACAAG-3′	5′-ATGTCACAGCCGAGATGG-3′
*scd1*	5′-GCAGGACGATATCTCTAGCT-3′	5′-GTCTCCAACTTATCTCCTCCATTC-3′
*atgl*	5′-GTGTCAGACGGCGAGAATG -3′	5′-TGGA GGGAGGGAGGGATG-3′
*hsl*	5′-TCAGTGTCTAGGTCAGACTGG-3′	5′-AGGCTTCTGTTGGGTATTGGA-3′
*fasn*	5′-TATGCTTCTTCGTGCAGCAGTT-3′	5′-GCTGCCACACGCTCCTCTAG-3′
*dgat*	5′-ATTGCTGGCTCATCGCTGT-3′	5′-GGGAAAGTAGTCTCGAAAGTAGC-3′
*18S*	5′-TTCGAACGTCTGCCCTATCAA-3′	5′-ATGGTAGGCACGGCGACTA-3′

*c/ebpβ*, CCAAT/enhancer-binding protein beta; *c/ebpα*, CCAAT/enhancer-binding protein alpha; *pparγ*, peroxisome proliferator fator gamma; *srebp1c*, sterol regulatory element-binding protein 1c; *acc*, acetyl-CoA carboxylase; *scd1*, stearoyl-CoA desaturase 1; *atgl*, adipose triglyceride lípase; *hsl*, hormone sensitive lípase; *fasn*, fatty acid synthase; *dgat*, diglyceride acyltransferase

PCR parameters were as follows: initial 2 min at 50 °C, denaturation at 95 °C for 10 min followed by 40 cycles of denaturation at 95 °C for 30 s, annealing at 60 °C, except 64.5 °C for *scd1* and 66.3 °C for *dgat*, for 30 s, and extension at 60 °C for 30 s. The results are expressed as fold changes of threshold cycle (Ct) value relative to controls using the 2^−ΔΔCt^ method [25].

### Cytotoxicity assay

Cell viability was assessed using the neutral red assay (TOX4 kit, Sigma-Aldrich, St. Louis, MO, USA) following manufacturer’s recommendations.

### Statistical analysis

Results are expressed as mean ± SEM. Statistical analysis was performed using SPSS 24.0 (SPSS, Chicago IL, USA). Comparisons between each treatment and the controls were carried out by Student’s *t* test. Statistical significance was set at the *P* < 0.05 level.

## Results

### Effect of apigenin, hesperidin and kaempferol on triacylglycerol content and cell viability in hMSC-derived adipocytes during differentiation

**Fig. 1:**
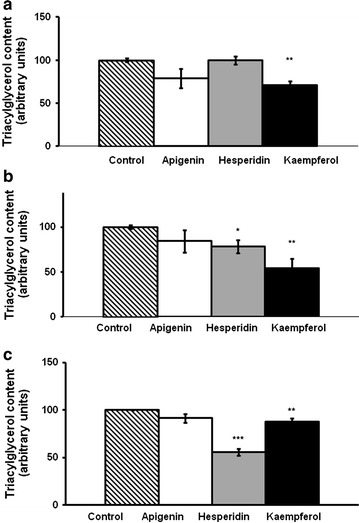
Triacylglycerol content in maturing pre-adipocytes derived from human mesenchymal stem cells treated with apigenin, hesperidin or kaempferol 1 µM (**a**), 10 µM (**b**) or 25 µM (**c**) or not (control cells) for 8 days. Values are mean ± SEM. Comparisons between each treatment and the control were analyzed by Student’s *t* test. Asterisks represent differences between phenolic compounds-treated cells and control cells. *P < 0.05; **P < 0.01; ***P < 0.001

**Fig. 2:**
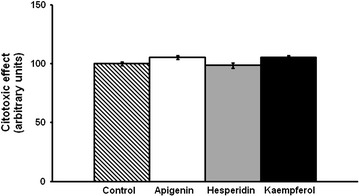
Cell viability in maturing pre-adipocytes derived from human mesenchymal stem cells treated with apigenin, hesperidin or kaempferol at 25 µM, or not (control cells) for 8 days. Values are mean ± SEM. Comparisons between each treatment and the control were analyzed by Student’s *t* test

While apigenin did not decrease TG content, hesperidin induced a decrease at both 10 and 25 µM and kaempferol at the three doses used (Fig. 1a–c). Apigenin, hesperidin or kaempferol did not produce a loss of viability of hMSCs even when exposed to the highest concentration of each compound (25 µM) (Fig. 2).

### Effect of apigenin, hesperidin and kaempferol in hMSC-derived adipocytes during differentiation

**Fig. 3:**
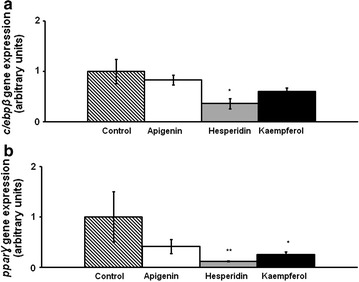
*c/ebpβ* (**a**) and *pparγ* (**b**) gene expression in maturing pre-adipocytes derived from human mesenchymal stem cells treated with apigenin, hesperidin or kaempferol at 25 µM or not (control cells) for 3 days. Values are mean ± SEM. Comparisons between each treatment and the control were analyzed by Student’s *t* test. *P < 0.05; **P < 0.01

On day 3, the expression of *c/ebpβ* and *pparγ* was measured in cells treated with the three phenolic compounds at 25 µM. Apigenin did not modify the expression of these genes. By contrast, hesperidin-treated cells showed lower mRNA levels of both genes. In the case of kaempferol, cells showed a significantly reduction in *pparγ* gene expression and a trend towards lower values in the case of *c/ebpβ* (*P* = 0.06) (Fig. 3).

**Fig. 4:**
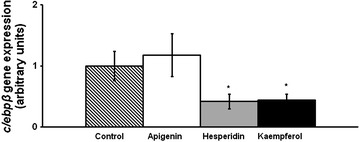
*c/ebpβ* gene expression in maturing pre-adipocytes derived from human mesenchymal stem cells treated with apigenin, hesperidin or kaempferol at 25 µM or not (control cells) for 8 days. Values are mean ± SEM. Comparisons between each treatment and the control were analyzed by Student’s *t* test. *P < 0.05

**Fig. 5:**
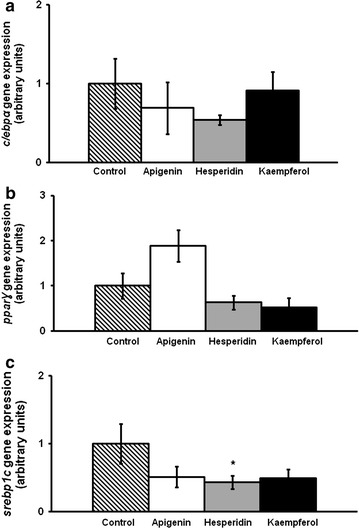
*c/ebpα* (**a**), *pparγ* (**b**) and *srebp1c* (**c**) gene expression in maturing pre-adipocytes derived from human mesenchymal stem cells treated with hesperidin or kaempferol at 25 µM or not (control cells) for 8 days. Values are mean ± SEM. Comparisons between each treatment and the control were analyzed by Student’s *t* test. *P < 0.05

**Fig. 6:**
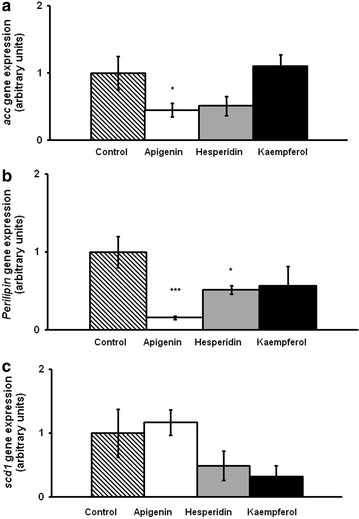
*acc* (**a**), *perilipin* (**b**) and *scd1* (**c**) gene expression in maturing pre-adipocytes derived from human mesenchymal stem cells treated with apigenin, hesperidin or kaempferol at 25 µM or not (control cells) for 8 days. Values are mean ± SEM. Comparisons between each treatment and the control were analyzed by Student’s *t* test. *P < 0.05; **P < 0.01

On day 8, apigenin did not modify *c/ebpβ* gene expression, while hesperidin and kaempferol-treated cells showed lower mRNA levels than the control cells (Fig. 4). Furthermore, gene expression of transcription factors involved in the intermediate stage of adipogenesis, *c/ebpα*, *pparγ and srebp1c*, is presented in Fig. 5. Only hesperidin significantly reduced *srebp1c* mRNA levels. Regarding the late stage of adipogenesis, *acc*, *perilipin* and *scd1* gene expression was determined. Apigenin reduced *acc* and *perilipin* gene expression. In turn, hesperidin induced a reduction in *perilipin* mRNA levels (Fig. 6).

### Effect of apigenin, hesperidin and kaempferol on triacylglycerol content in mature adipocytes derived from hMSCs

**Fig. 7:**
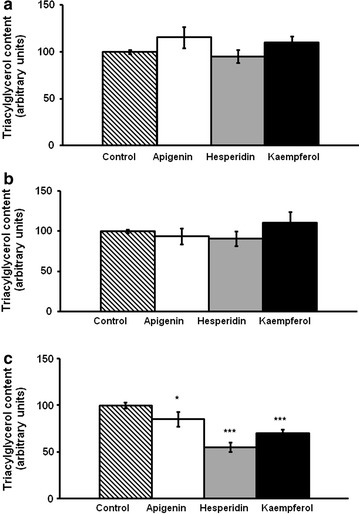
Triacylglycerol content in mature adipocytes derived from human mesenchymal stem cells treated with apigenin, hesperidin or kaempferol 1 µM (**a**), 10 µM (**b**) or 25 µM (**c**) or not (control cells) for 48 h. Values are mean ± SEM. Comparisons between each treatment and the control were analyzed by Student’s t test. Asterisks represent differences between phenolic compounds-treated cells and control cells. *P < 0.05; **P < 0.01; ***P < 0.001

The lowest doses, 1 and 10 µM of apigenin, hesperidin or kaempferol did not reduce TG content in mature adipocytes (Fig. 7a and b). However, 25 µM of the three phenolic compounds led to a significant diminution of TG (Fig. 7c).

### Effect of apigenin, hesperidin and kaempferol on gene expression of mature adipocytes derived from hMSCs

**Fig. 8:**
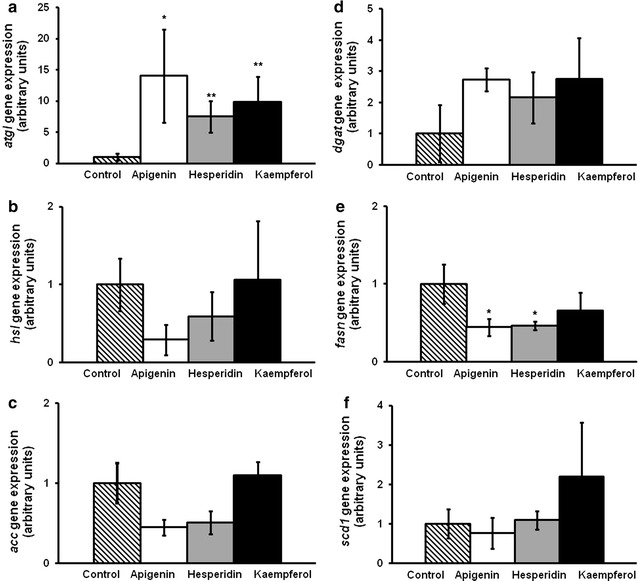
*Atgl* (**a**), *hsl *(**b**), *acc* (**c**), *fasn *(**d**), *dgat* (**e**) and *scd1* (**f**) gene expression in mature adipocytes derived from hMSCs17 stem cells treated with hesperidin or kaempferol at 25 µM or not. Values are mean ± SEM. Comparisons between each treatment and the control were analyzed by Student’s *t* test. *P < 0.05; **P < 0.01

Gene expression in mature adipocytes was measured at 25 µM. The three compounds increased *atgl* mRNA levels (Fig. 8a). In addition, apigenin and hesperidin decreased gene expression of *fasn* (Fig. 8e). No changes were observed in the expression of *hsl, acc*, *dgat2* and *scd1* (Fig. 8b–d, f).

## Discussion

In recent years, a large number of scientific studies have focused on phenolic compounds as potential new tools for obesity management. In this context, our group previously analyzed the effect of fifteen phenolic compounds, belonging to different chemical groups, on 3T3-L1 pre-adipocytes, in order to know the potential relationship between the efficacy on adipogenesis inhibition and the chemical structure [23].

The present work aimed to study the responses of human pre-adipocytes and adipocytes obtained from stem cells to three polyphenols: (a) apigenin, a natural flavonoid widely distributed in plant foods such as chamomile tea, grapefruit, onions, oranges and some spices like parsley, (b) hesperidin, a flavanone glycoside abundant in citrus fruits and (c) kaempferol, a natural flavonol originally isolated from tea, broccoli and other plant sources, and to compare them with those obtained previously in murine adipocytes under the same experimental conditions. We selected these three compounds because they showed greater effectiveness in our previous study carried out in 3T3-L1 [18] and because their mechanisms of action have been little studied. We carried out the experiment in a range from a physiological dose of 1 μM [26–32] to a high dose of 25 μM.

Apigenin did not show an anti-adipogenic action. By contrast, pre-adipocytes treated with hesperidin and kaempferol showed reduced TG content at the end of the maturation process, suggesting that these compounds induced a reduction in adipogenesis. These results are different from those obtained in our laboratory in 3T3-L1 pre-adipocytes [23]. In these cells, apigenin and kaempferol were anti-adipogenic at 10 and 25 μM, and hesperidin was effective at the three experimental doses. These results suggest that kaempferol, and mainly hesperidin, could be useful to prevent obesity in those stages of life where adipogenesis, significantly contributes to obesity development, such as childhood, adolescence and adults with severe levels of obesity. By comparing these results with those previously observed in 3T3-L1 pre-adipocytes, we can state that the latter seem to be more responsive to apigenin and hesperidin than pre-adipocyte derived from human stem cells, but not to kaempferol. This confirms the existence of important interspecies differences in adipocyte function and thus the difficulty in extrapolating results from murine adipocytes to human adipocytes.

In order to discard a potential involvement of cytotoxic effects in adipogenesis reduction, we measured cell viability when cells were incubated with the highest dose (25 μM). The study showed that the molecules did not decrease cell viability. These results are in good accordance with Morikawa et al. [33] who reported no cytotoxic effect for hesperidin at 500 μM in adipocytes derived from human bone marrow stromal cells. Other authors have performed experiments treating hMSCs at different doses, not using isolated phenolic compounds but plant extracts containing kaempferol, hesperidin and apigenin among other phenolic compounds [34, 35]. The only toxic effect was observed at the highest doses used (above 0.5 mg/mL). The authors concluded that this effect might be due to synergism between compounds present in the plant extract.

One of the objectives of the present study was to elucidate the mechanism by which apigenin, hesperidin and kaempferol reduced adipogenesis, and for that purpose the expression of genes involved in this pathway was analyzed. Three phases can be distinguished in adipogenesis: early, intermediate and late [36]. During clonal expansion, there is an induction of the early phase transcription factor, *c/ebpβ*, which leads to transactivation of the transcription factors of the intermediate stage *pparγ*, c*/ebpα* and *srebp1c* [36]. During the late phase of differentiation, adipocytes markedly increase de novo lipogenesis and acquire sensitivity to insulin. The mRNA levels of enzymes involved in TG metabolism, including *acc* and *scd1* among others, increase 10–100 fold. The transcription factors of the intermediate stage are implicated in the activation of these last genes [37].

Under our experimental conditions, apigenin did not modify the expression of the main adipogenic genes (*c/ebpβ*, *c/ebpα*, *pparγ* and *srebp1c*) [38]. This is in good accordance with the observed lack of reduction in TG content. As far as we know, there are no studies in the literature on human adipocytes derived from stem cells treated with this phenolic compound, and thus comparisons cannot be made. By contrast, in 3T3-L1 pre-adipocytes many authors analyzed the anti-adipogenic effect of this phenolic compound. Kim et al. [39], observed that 70 μM apigenin reduced cell TG content by modulating the expression of the adipogenic transcriptional factors C/EBPβ, PPARγ and C/EBPα. These results are in good accordance with that of Ono et al. [40], who observed an anti-adipogenic effect of apigenin at 10 and 50 μM, but not at 1 mM. In our laboratory we analyzed the effect of this compound on 3T3-L1 cells in the same experimental conditions, and we observed reductions in TG content when incubations were carried out with 10 and 25 μM [23]. Other authors did not observe a reduction in TG content in the same cells at a dose of 10 μM [41].

Hesperidin inhibited genes involved in the three phases of adipogenesis, *c/ebpβ srebp1c*, *pparγ* and *perilipin*. These results justify the reduction in TG content induced by this phenolic compound. To the best of our knowledge, there are no in vitro studies analyzing the anti-adipogenic effect of pure hesperidin in pre-adipocytes derived from hMSCs. There is a study performed with an extract of *Citrus bergamia*, which contains 13% hesperidin, where hMSCs treated during differentiation showed a significant reduction in TG accumulation and *pparγ* expression [34]. Nevertheless, the comparison with our results is difficult because the amount of hesperidin provided to the cells in that study is far lower than that used in our experiment. In addition, it contains other phenolic compounds that may also have an effect on pre-adipocytes and interact among themselves. In 3T3-L1 pre-adipocytes, Jeon et al. [42], in good accordance with our results, observed a delipidating effect in pre-adipocytes treated with 10 and 20 μM hesperidin. Moreover, in our laboratory we observed a decrease in TG content in this type of cells when they were incubated for 8 days with 1, 10 or 25 μM. The effect was mediated by a decrease in *srebp1c* [23].

As far as kaempferol-treated cells are concerned, despite only *c/ebpβ* and *pparγ* being significantly reduced, an anti-adipogenic effect was observed, suggesting that the inhibition of genes involved in the early and intermediate stages of adipogenesis was enough to reduce this process. In fact, a similar situation took place in a previous study from our laboratory when 3T3-L1 pre-adipocytes were incubated with resveratrol or some of its metabolites [15]. As far as we are aware, there are no studies in the literature that demonstrate the anti-adipogenic effect of this compound in adipocytes derived from hMSCs. In 3T3-L1 pre-adipocytes, doses higher than 5 μM of kaempferol exerted a significant anti-adipogenic effect [43]. Moreover, it was observed that also at 40 μM, this compound decreased TG accumulation by the down-regulation of *pparγ* and *srebp1c* [44]. In our studies performed in this type of cells, kaempferol reduced TG content at 10 and 25 μM, but not at 1 μM. The effect was mediated by a decrease in *c/ebpβ* and *srebp1c* [23].

The present study also aimed to determine the effect of the three phenolic compounds in mature adipocytes. All of them reduced TG accumulation at the dose of 25 µM after 48 h of treatment, but not at lower doses. Taking into account that 25 µM is far higher than the serum concentrations and tissue amounts found in animals treated with polyphenols, we may say that in all likelihood these phenolic compound are not useful to reduce body fat accumulation in adult humans. Unfortunately, there are no studies in the literature showing the effects of pure apigenin, hesperidin or kaempferol on mature adipocytes derived from hMSCs. As far as plant extracts containing these phenolic compounds are concerned, Lo Furno et al. [34] showed that treating hMSCs cells with 10 or 100 µg/mL of *C. bergamia* extract during 7 or 14 days reduced TG accumulation. Colitti et al. [45] demonstrated that a plant extract from *Citrus aurantium,* containing hesperidin, reduced lipid accumulation in primary human mature adipocytes.

When the expression of genes involved in TG metabolism of mature adipocytes was analyzed, all compounds increased mRNA levels of a very well known lipase, *atgl*. By contrast, *hsl* remained unchanged. This could be explained by the fact that *hsl* mediates lipolysis stimulated by catecholamines and by natriuretic peptide, whereas *atgl* mediates the hydrolysis of TG during basal lipolysis [46]. Moreover, apigenin and hesperidin decreased gene expression of *fasn*. These results as a whole suggest that while the three compounds reduced TG content in mature human adipocytes by affecting, at least in part, the lipolytic process, in the case of apigenin the decrease in the lipogenic pathway also contributed to the delipidating effect.

This is the first time that the effect of pure apigenin, hesperidin and kaempferol has been tested in mature adipocytes derived from hMSCs. However, in the study of Lo Furno et al. [34], where *C. bergamia* extracts were used to treat hMSCs for 14 days, the authors observed that 10 and 100 µg/mL of the plant extract increased lipase protein levels, results that are in line with those of the present study [27]. As explained previously in this manuscript, a comparison with our results is difficult because of the presence of other active phenolic compounds and the potential synergism.

## Conclusions

In summary, the present study shows the anti-adipogenic and delipidating effects of apigenin, hesperidin and kaempferol in human adipocytes derived from hMSCs for the first time. While hesperidin and kaempferol reduce adipogenesis, apigenin was ineffective. Regarding mature adipocytes, the three compounds (hesperidin, kaempferol and apigenin) reduce TG accumulation by activating, at least in part, lipolysis, and in the case of hesperidin and apigenin, also by reducing lipogenesis. Nevertheless, doses higher than those found in serum and plasma of animals treated with polyphenols are needed to show the delipidating effect in mature adipocytes.

## Abbreviations

*acc*: acetyl-CoA carboxylase
*atgl*: adipose triglyceride lipase
Ct: threshold cycle
*dgat*: diglyceride acyltransferase
*c/ebpα*: CCAAT/enhancer-binding protein alpha
*c/ebpβ*: CCAAT/enhancer-binding protein beta
*fasn*: fatty acid synthase
FBS: fetal bovine serum
*hsl*: hormone sensitive lipase
MSC: mesenchymal stem cells
PBS: phosphate buffer saline
*pparγ*: peroxisome proliferator factor gamma
*scd1*: stearoyl-CoA desaturase 1
*srebp1c*: sterol regulatory element-binding protein 1c
TG: triacylglycerol
